# Non-Coding Negative Regulatory Features in Livestock Genomes: Functional Annotation and Causal Validation

**DOI:** 10.3390/ani16182956

**Published:** 2026-09-20

**Authors:** Lingzehui Zhang, Bingjie Ma, Yiming Zhang, Xin Wang, Le Wang, Ruijun Wang, Yanjun Zhang, Fangzheng Shang

**Affiliations:** 1 College of Animal Science, Inner Mongolia Agricultural University, Hohhot 010018, China; 2 Key Laboratory of Meat Sheep Genetic Breeding, Ministry of Agriculture and Rural Affairs, Hohhot 010018, China; 3 Key Laboratory of Sheep Genetic Breeding and Reproduction of Inner Mongolia Autonomous Region, Hohhot 010018, China

**Keywords:** negative regulatory features, silencers, functional genomics, epigenetic regulation, causal validation, livestock

## Abstract

Non-coding negative regulation is an important but incompletely resolved component of gene-expression control in livestock. We conducted a narrative review using PubMed, Web of Science Core Collection, and Scopus, prioritizing primary studies in pigs, cattle, sheep, goats, and poultry and using model-organism studies only for directly relevant mechanistic or methodological evidence. We distinguish sequence-defined silencers, context-dependent enhancer-silencer elements, functional microRNA-binding sites, long non-coding RNA-associated regulation, and candidate repressive chromatin regions, and evaluate representative livestock studies using a four-tier framework comprising candidate annotation, molecular functional evidence, endogenous causal evidence, and animal-level validation. Current evidence is strongest for a small number of loci, whereas many of the livestock candidates reviewed here are supported primarily by association, chromatin state, or in vitro assays. We further assess how multi-omics data can prioritize candidates and assign target genes, while emphasizing that reporter activity, repressive chromatin marks, and population association do not establish endogenous causality. Progress will depend on locus-specific perturbation, rescue experiments, relevant animal phenotyping, and inheritance testing. This evidence-centered framework provides a focused basis for interpreting negative regulatory variation in livestock and identifying robust targets for future genetic improvement.

## Simple Summary

Genes are controlled not only by DNA regions that switch them on but also by features that reduce or restrict gene activity. In farm animals, these negative regulatory features may influence growth, milk production, reproduction, and embryo development. However, many candidate negative regulatory features are identified only because they occur near a trait-associated variant or in a region with molecular signs of gene repression. Such associations do not prove that the region directly controls a gene. This review organizes the main types of negative regulation and compares the evidence needed to move from a candidate region to direct functional testing and, ultimately, confirmation in animals and their offspring. Many livestock examples still lack this complete chain of evidence. Clearer standards and targeted experiments should help researchers distinguish causally supported negative regulatory features from correlated signals and identify variants that can be used more reliably to understand biology and improve livestock breeding.

## 1. Introduction

Complex traits in livestock, including growth, reproduction, meat quality, and lactation, are influenced by non-coding regulatory variation. Advances in functional genomics have enabled extensive annotation of promoters, enhancers, and trait-associated regulatory regions in livestock [1,2]. However, most functional annotation efforts have focused on activating regulatory elements, whereas negative regulatory features remain less systematically characterized.

Negative regulatory features are particularly difficult to identify because they lack universal sequence or chromatin signatures and frequently depend on cell type, developmental stage, and environmental context [3,4,5]. Some silencers occur in relatively inaccessible chromatin, whereas others remain accessible to repressive transcription factors, and the same DNA sequence may even display activating or repressive activity in different contexts. Consequently, association signals, repressive chromatin states, or reduced reporter activity can nominate candidate regions but do not by themselves establish endogenous negative regulatory function. This gap between candidate annotation and causal validation remains a major limitation in livestock regulatory genomics.

In this review, “negative regulatory features” is used as an umbrella term encompassing (i) sequence-defined transcriptional negative regulatory elements, including silencers and context-dependent enhancer-silencer elements; (ii) non-coding RNA-associated negative regulation, including functional microRNA (miRNA)-binding sites and long non-coding RNA (lncRNA)-associated mechanisms [6]; and (iii) candidate repressive chromatin regions characterized by DNA methylation or repressive histone modifications. These classes differ in molecular substrate, regulatory level, and the experimental evidence required to establish causality.

Previous livestock reviews have largely emphasized epigenomic annotation, prioritization of functional variants, or applications of genome-editing technologies [7,8]. A distinct unresolved issue is how to determine whether a candidate locus is itself a negative regulatory feature rather than a correlated chromatin state or a linked marker. This review therefore focuses on two questions: how negative regulatory features should be defined across different molecular classes, and what evidence is required to progress from candidate annotation to endogenous and animal-level causality. We use a four-tier framework spanning candidate annotation, molecular functional evidence, endogenous causal evidence, and animal-level causal evidence, and apply it to representative livestock studies. The aim is not to catalog genome-wide association signals or editing platforms, but to evaluate the evidentiary chain linking a candidate feature to a target gene, a directional molecular effect, and a biologically relevant livestock phenotype.

## 2. Methods

This narrative review was based on searches of PubMed, Web of Science Core Collection, and Scopus. The literature search was last updated on 4 September 2026. The same four conceptual search themes were applied across databases: (1) “negative regulatory elements” OR “silencers”; (2) “miRNA-binding sites”; (3) “lncRNA-associated negative regulation”; and (4) “repressive chromatin” OR “epigenetic repression”. Each theme was combined with livestock-related terms, including cattle, cow, bovine, pig, swine, sheep, ovine, goat, caprine, chicken, and poultry. Database-specific search themes and the numbers of records retrieved are summarized in Table 1. The complete database-specific search strategies, including Boolean operators and search fields, are provided in Supplementary Table S1.

Primary studies in pigs, cattle, sheep, goats, and poultry were prioritized as the direct evidence base for this review. Evidence from human and model-organism studies was used only to illustrate conserved mechanisms, conceptual principles, or methodological approaches relevant to the interpretation and validation of negative regulatory features. Such evidence was not weighted equivalently to direct livestock evidence and was not used to increase the evidence-tier assignment of livestock loci. Evidence tiers were assigned according to the level of functional and causal evidence directly demonstrated in livestock studies. Records from the three databases were combined, and duplicates were removed before screening. Records were screened first by title and abstract and then assessed for eligibility. Studies were excluded when they addressed only protein-coding gene knockout, non-locus-specific global transcriptional repression, or reduced expression without locus-level regulatory evidence. Reviews were used for conceptual background and to identify additional primary studies. Additional details of the study-selection process are provided in Supplementary Table S2. Representative livestock studies discussed in detail were selected to illustrate the major regulatory classes and different levels of functional evidence, with priority given to studies providing locus-level evidence, explicit target-gene relationships, endogenous perturbation, and/or animal phenotypes; these examples are illustrative rather than comprehensive. The eligibility and representative-study selection criteria are provided in Supplementary Table S3. As this was a narrative review, no quantitative meta-analysis was performed.Across the three databases, 2363 records were identified (PubMed, 545; Web of Science, 765; Scopus, 1053). After removal of 1169 duplicate records, 1194 unique records remained for title and abstract screening. A total of 556 records were excluded at this stage, 638 articles were assessed for eligibility, 278 were excluded during eligibility assessment, and 360 studies were retained for the final narrative synthesis. The study-selection stages are summarized in Table 2.

## 3. Conceptual Framework and Evidence Standards for Negative Regulatory Features

### 3.1. Conceptual Scope and Classification Boundaries

**Figure 1:**
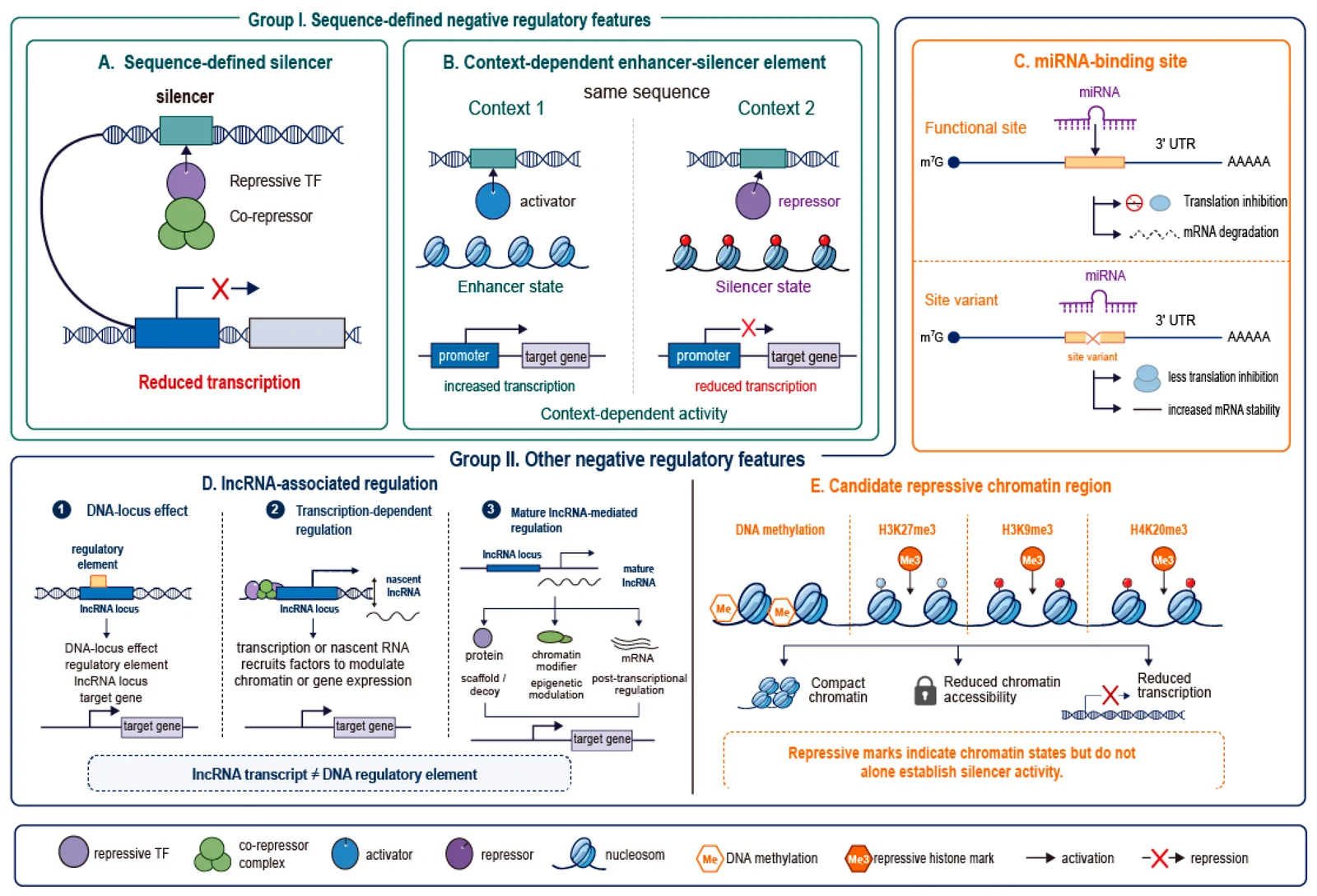
Major classes and mechanistic distinctions of negative regulatory features in livestock genomes.

Negative regulatory features encompass mechanistically distinct forms of non-coding regulation and are grouped here for comparative evaluation of evidence rather than because they share a common molecular mechanism. Sequence-defined transcriptional negative regulatory elements include silencers and context-dependent enhancer-silencer elements, both of which act through defined DNA sequences but may differ in their context dependence and regulatory direction [9]. Non-coding RNA-associated negative regulation operates through a different molecular layer: miRNA-binding sites mediate post-transcriptional repression, whereas lncRNA-associated effects may arise from the genomic locus, the act of transcription, or the mature RNA molecule. Candidate repressive chromatin regions form a third category. DNA methylation and repressive histone modifications such as H3K27me3, H3K9me3, and H4K20me3 describe chromatin states that may accompany or contribute to transcriptional repression, but they do not by themselves define an independently acting negative regulatory element. Accordingly, evidence from these mechanistically distinct categories is evaluated using category-specific functional criteria while being compared within the common evidence-tier framework. Their mechanistic distinctions are illustrated in Figure 1, and the minimum evidence required for functional assignment is summarized in Table 3.

### 3.2. Negative Regulatory Elements with Defined Sequence Boundaries

Silencers are cis-regulatory DNA sequences that reduce target-gene transcription in a context-dependent manner and may occur near promoters, within introns, or in distal intergenic regions [9]. Mechanistic evidence from human and mouse systems has shown that silencer activity can vary substantially across cell types and chromatin contexts and that some Polycomb repressive complex 2 (PRC2)-bound distal regions can repress developmental genes through spatial interactions with target promoters [10,18,19,20]. These studies establish important mechanistic principles for silencer biology; however, direct livestock evidence for comparable sequence-defined mechanisms remains more limited.

The regulatory direction of a DNA element is also not necessarily fixed. In Drosophila, some transcriptional silencers can function as enhancers in alternative cellular contexts [11]. This model-organism evidence supports the general concept of context-dependent enhancer–silencer activity. However, equivalent endogenous evidence remains limited in livestock. Accordingly, candidate livestock elements should be classified as context-dependent enhancer-silencer elements only when their regulatory direction is demonstrated in the relevant tissue, developmental stage, or physiological context.

### 3.3. Non-Coding RNA-Associated Negative Regulation

miRNA-binding sites are located predominantly in the 3′ untranslated regions (UTRs) of target mRNAs and influence mRNA stability or translational efficiency through pairing between the miRNA seed region and complementary target sequences [21]. Single-nucleotide polymorphisms (SNPs), insertions and deletions (InDels), and haplotype differences within these sites may strengthen or weaken miRNA binding and thereby alter post-transcriptional repression. Because miRNA targeting is influenced not only by seed pairing but also by local RNA structure, supplementary base pairing, and cellular context, computational prediction, negative expression correlation, or co-expression analysis alone cannot establish a direct miRNA-target interaction.

Functional validation of an miRNA-binding site should include allelic or site-directed mutagenesis, manipulation of miRNA abundance, and measurement of reporter activity or endogenous target-protein expression. A site should be considered a functional post-transcriptional negative regulatory feature only when the specific binding sequence is identified, and alteration of that sequence is shown to change miRNA-mediated repression.

lncRNA-associated negative regulation is more complex because the functional effect may arise from DNA regulatory sequences within the lncRNA locus, transcription through the region, or the mature lncRNA molecule [14,15,22]. Some lncRNAs act in cis on nearby genes, whereas others act in trans by interacting with DNA, RNA, or proteins at distant loci. lncRNAs can also influence chromatin folding, transcription-factor recruitment, mRNA stability, splicing, and translation.

An lncRNA transcript should therefore not be equated directly with a non-coding DNA regulatory element. Deletion of an lncRNA promoter may simultaneously disrupt local DNA regulatory sequences and the transcription process, whereas small interfering RNA (siRNA) or antisense-oligonucleotide depletion of the mature RNA cannot determine whether the genomic locus itself has a cis-regulatory role. For high-priority lncRNA candidates, the genomic locus, transcriptional elongation, and mature RNA molecule should be perturbed separately to identify the molecular layer responsible for negative regulation.

### 3.4. Candidate Repressive Chromatin Regions

DNA methylation and histone modifications such as H3K27me3, H3K9me3, and H4K20me3 are frequently associated with reduced transcriptional activity. DNA methylation can alter transcription-factor binding, recruit methyl-CpG-binding proteins, and modify local chromatin structure. H3K27me3 deposited by Polycomb repressive complexes contributes mainly to developmentally regulated gene silencing, whereas H3K9me3 and H4K20me3 are commonly associated with stable heterochromatin and long-term repression [23,24].

Repressive histone modifications and DNA methylation often span broad chromatin domains whose boundaries do not coincide with individual cis-regulatory elements. High methylation or enrichment of repressive histone marks therefore does not demonstrate that a region functions as an autonomous silencer. Such states may cause reduced transcription, but they may also arise as a consequence of transcriptional shutdown. Differentially methylated regions and regions enriched for repressive histone modifications should initially be classified as candidate repressive chromatin regions. Causality requires targeted methylation or demethylation, epigenome editing, perturbation of the relevant modifying enzymes, or mutation of critical sequences, followed by assessment of endogenous target-gene expression.

### 3.5. Functional Evidence Tiers

To reduce subjectivity in the evaluation of negative regulatory features, we refined the four-tier evidence framework using explicit operational criteria, informed by studies of silencer identification, functional perturbation, and epigenome editing [9,10,16,17,18]. The tiers describe the highest level of evidence directly supported by a study rather than an obligatory sequence through which every candidate must progress. Evidence from a lower tier alone does not justify automatic progression. In particular, molecular activity demonstrated in reporter, binding, or interaction assays (Tier 2) does not establish endogenous causality, which requires locus- or mechanism-specific perturbation in the relevant endogenous context (Tier 3). Animal-level causal evidence (Tier 4) additionally requires that the validated regulatory perturbation or naturally occurring causal allele be linked to a relevant phenotype in livestock.

The operational criteria are applied according to the molecular class of the regulatory feature. Thus, the specific experimental requirements for a sequence-defined silencer, miRNA-binding site, lncRNA-associated mechanism, or candidate repressive chromatin region may differ, although the evidentiary distinction between candidate annotation, molecular function, endogenous causality, and animal-level causality remains the same. Each study is assigned to the highest tier for which all defining minimum criteria are satisfied. Failure to satisfy a higher-tier criterion does not imply that the proposed biological mechanism is absent; it indicates only that the available evidence does not establish that level of causality.The operational definitions, minimum criteria for assignment, and representative approaches for the four evidence tiers are summarized in Table 4.

The Functional Annotation of Animal Genomes (FAANG) initiative and subsequent studies have generated transcriptomic, chromatin-accessibility, and histone-modification maps for domesticated species including pigs, cattle, and chickens, providing an essential foundation for livestock regulatory annotation [25,26]. The Farm Animal Genotype-Tissue Expression (FarmGTEx) project further integrates multi-tissue gene expression, genetic variation, and regulatory effects, thereby extending the analysis of regulatory variation to the population level [27]. These resources substantially improve candidate discovery, but relatively few negative regulatory loci have progressed to endogenous perturbation and animal-level phenotyping.

## 4. Negative Regulatory Features Associated with Complex Traits in Livestock

Negative regulation in non-coding regions does not necessarily have an adverse effect on production traits. Silencers, miRNA-binding sites, lncRNA-associated regulation, and repressive chromatin states can maintain normal development and physiological homeostasis by restricting the level, timing, and tissue distribution of gene expression. Effects on growth, meat quality, lactation, reproduction, or embryonic development arise when natural variation, epigenetic change, or experimental perturbation disrupts this regulatory balance. The depth of evidence varies widely among studies, from multi-omics annotation and population association to in vitro functional assays, endogenous genome editing, and animal phenotyping. This section summarizes representative studies by trait and biological process and evaluates them using the evidence framework introduced above.

### 4.1. Growth and Body Composition

Growth, muscle development, and fat deposition currently provide some of the strongest livestock examples of negative regulation. Relevant loci can influence body weight, lean yield, and meat quality by altering growth-factor dosage, miRNA-mediated post-transcriptional repression, or the differentiation of muscle and adipose cells.

The g.8795C>T variant in the 3′ UTR of *PLAG1* in Hu sheep alters binding of miR-139. Population analyses associated this locus with birth and weaning weights, while dual-luciferase assays and miR-139 gain- and loss-of-function experiments indicated that the T allele weakens miR-139-mediated repression and increases *PLAG1* expression [28]. Accordingly, this locus is classified as Tier 2, although endogenous allele replacement is still required to distinguish its effect from that of linked variation.

The ZBED6-binding motif in intron 3 of porcine *IGF2* is supported by a more complete causal evidence chain. Clustered regularly interspaced short palindromic repeats (CRISPR)/CRISPR-associated protein 9 (Cas9)-mediated disruption of the motif relieves ZBED6-dependent transcriptional repression, increases *IGF2* expression, and enhances muscle development in edited pigs [29]. Subsequent cytosine base editor 3 (CBE3)-mediated conversion of C3071 to T altered muscle growth, carcass composition, intramuscular fat, and several meat-quality traits in edited pigs and their offspring [30]. This locus is supported by endogenous editing, animal phenotyping, and offspring validation and represents one of the best-characterized sequence-defined negative regulatory features in livestock. Its breeding value nevertheless requires evaluation in larger populations and across breeds, together with assessment of potential tissue-specific consequences of altered *IGF2* dosage.

A genome-wide atlas of porcine repressor elements has identified numerous candidate regions associated with muscle, adipose, and metabolic traits [31]. Functional-genomic and comparative-epigenomic maps in chickens, sheep, and other ruminants likewise show that trait-associated regulatory regions are highly tissue- and species-specific [32,33,34]. These candidates currently remain at Tier 1.

### 4.2. Mammary Gland Development and Lactation

Lactation and milk-protein traits are influenced by mammary development, milk-protein synthesis, endocrine signaling, and post-transcriptional regulation. Polymorphisms in the 3′ UTR of bovine *CSN3* alter the binding of bta-miR-708 and affect *CSN3* mRNA and κ-casein expression [35]. Reporter assays together with mRNA and protein measurements support a role for this region in miRNA-mediated post-transcriptional regulation.

The major production traits evaluated in that study did not, however, show consistent significant differences, indicating that a molecular expression effect does not necessarily translate into a detectable lactation phenotype. The locus therefore provides Tier 2 molecular functional evidence but cannot yet be regarded as a breeding target with a defined production effect. Replication across dairy populations, lactation stages, and genetic backgrounds, together with endogenous allele replacement followed by evaluation of milk-protein composition, is still required.

Integrated analysis using assay for transposase-accessible chromatin sequencing (ATAC-seq) and RNA sequencing (RNA-seq) of porcine mammary placodes identified candidate open-chromatin regions and genes associated with teat number and mammary development [36]. In the absence of endogenous sequence perturbation, these findings remain Tier 1 candidate annotation evidence.

### 4.3. Ovarian Function and Follicular Dynamics

Follicle recruitment, granulosa-cell proliferation, follicular atresia, and ovulation all require stage-specific restriction of gene expression. DNA methylation, repressive histone modifications, and non-coding RNAs can contribute to these processes, although individual studies often resolve different molecular layers of regulation.

Integrated methylome and transcriptome analyses of ovaries from high- and low-prolificacy Hu sheep identified candidate differentially methylated regions associated with ovarian function [37]. Because their regulatory effects have not been tested by targeted methylation perturbation, these regions remain Tier 1 candidates.

In porcine granulosa cells, H3K27me3 enrichment at the *FOXO1* promoter was associated with regulation of *FOXO1* and its downstream target *CYP1A1*, with consequences for granulosa-cell proliferation and follicular development [38]. The combination of chromatin profiling, expression perturbation, and cellular phenotyping supports Tier 2 molecular functional evidence. Importantly, the study defines an epigenetic regulatory axis rather than a sequence-bounded cis-silencer.

Studies of porcine follicular atresia further showed that the antisense lncRNA *BRE-AS* contributes to granulosa-cell apoptosis [39]. RNA-interference experiments support a cellular function for *BRE-AS*, but they do not distinguish whether the effect arises from the mature RNA, the transcription process, or DNA regulatory sequences within the locus. The locus should therefore not be classified as an lncRNA-associated negative regulatory feature solely on the basis of lncRNA expression changes.

Overall, reproduction-related studies have identified many differentially methylated regions, histone-modification sites, and lncRNA candidates, but few have progressed to endogenous locus perturbation and validation of reproductive phenotypes in animals.

### 4.4. Embryonic Development and Epigenetic Reprogramming

Embryonic and germ-cell development involve extensive DNA-methylation reprogramming, histone-modification transitions, and imprinting control. Genome-wide analyses of the porcine germline have revealed dynamic DNA-methylation changes during fetal development, demonstrating that the epigenetic state of a given genomic region can shift markedly across germ-cell stages [40]. Such maps provide a basis for identifying stage-specific candidate repressive chromatin regions but do not establish that any individual region has autonomous negative regulatory activity.

In porcine parthenogenetic embryos, depletion of *IDH2* and *GLUD1* reduced α-ketoglutarate levels and caused abnormal persistence of H4K20me3, thereby suppressing genes involved in embryonic genome activation and impairing development [41]. Perturbation and rescue experiments strengthened the causal evidence for this metabolic-histone-modification axis. Its effect, however, reflects a broad epigenetic state rather than a discrete cis-regulatory element.

Histone lactylation in early bovine embryos changes dynamically across developmental stages, and both excessive depletion and excessive accumulation impair embryonic genome activation and blastocyst formation [42]. These findings illustrate that the biological effects of histone modifications are dose- and stage-dependent and cannot be classified as activating or repressive solely from a unidirectional change in abundance.

The imprinted porcine lncRNA *KCNQ1OT1* binds the *CDKN1C* promoter region and influences local chromatin folding. Antisense-oligonucleotide-mediated depletion of *KCNQ1OT1* increased *CDKN1C* expression, supporting its involvement in cis-repression [43]. Because antisense oligonucleotides primarily target the RNA product, this experiment does not resolve the contribution of the *KCNQ1OT1* DNA locus or the act of transcription. Promoter replacement, premature transcriptional termination, and selective degradation of the mature RNA will be required to distinguish these mechanisms.

Taken together, studies of embryonic development have mainly revealed dynamic epigenetic regulatory processes; sequence-defined negative regulatory elements validated at their endogenous loci remain uncommon.

### 4.5. Evidence Comparison of Illustrative Cases

An additional bovine example is the lncRNA ADNCR, which suppresses adipogenic differentiation through miR-204-associated regulation [44]. The cases in Table 5 are illustrative rather than comprehensive and include both studies discussed above and additional livestock examples selected to broaden species, trait contexts, and regulatory mechanisms. Each study is assigned to the highest tier for which all defining minimum criteria are satisfied, based on the evidence reported in the original study rather than on the biological plausibility of the proposed mechanism.

Across these illustrative livestock studies, evidence is concentrated mainly at Tier 1 and Tier 2, with comparatively few examples providing the endogenous perturbation, animal phenotyping, and inheritance data required for Tier 3 or Tier 4 assignment.

## 5. Discovery and Prioritization: Supporting Evidence, Not Causality

Discovery approaches integrate genetic, epigenomic, transcriptomic, and spatial information to prioritize candidate negative regulatory features for subsequent functional testing [27,54,55,56].

### 5.1. Genetic and Epigenomic Candidate Discovery

Genome-wide association studies (GWAS) and quantitative trait locus (QTL) mapping identify trait-associated regions, whereas molecular QTL analyses can connect alleles with gene expression or chromatin accessibility [27,57]. GWAS and molecular QTL signals can therefore be used to prioritize candidate negative regulatory features for functional testing. Representative livestock studies show how transcriptomic and regulatory-region signals can nominate candidates for meat quality, reproduction, and mammary development [36,39,58]. Chromatin-accessibility, DNA-methylation, and histone-modification maps add regulatory context [57,59,60], while conservation, transcription-factor motifs, and sequence-based prediction models can further rank candidates [61,62,63,64,65]. Low accessibility, DNA methylation, or a repressive histone mark should therefore be interpreted as supporting context rather than proof of silencer activity.

### 5.2. Target-Gene Assignment

A central difficulty is assigning distal regulatory candidates to the correct target gene. Linear proximity alone is insufficient because regulatory elements can bypass nearby genes. Chromosome-conformation methods, including high-throughput chromosome conformation capture (Hi-C), Hi-C coupled with chromatin immunoprecipitation (HiChIP), proximity ligation-assisted chromatin immunoprecipitation sequencing (PLAC-seq), and targeted chromosome conformation capture (Capture-C), provide spatial evidence, while expression and chromatin-accessibility QTL and allele-specific measurements provide population- and allele-level support [27,55,66]. The Activity-by-Contact (ABC) model can rank candidate target genes, but its use for negative regulation requires direct validation [67,68].

Target-gene assignment is strongest when sequence variation, factor binding, chromatin state, physical contact, and expression change converge on the same regulatory relationship. In chickens, a non-coding variant associated with abdominal fat deposition altered nuclear factor kappa B (NF-κB) binding and *BMP2* expression, illustrating how multiple lines of evidence can support a biologically relevant variant-target connection [69]. Comparative epigenomic studies further emphasize that such evidence should be interpreted within tissue- and species-specific contexts [70].

### 5.3. Cell-Type and Spatial Specificity

Bulk RNA sequencing, chromatin-accessibility profiling, and chromatin immunoprecipitation average signals across mixed cell populations and may obscure regulatory features that operate in rare or transient states. Single-cell transcriptomic and chromatin-accessibility approaches, together with spatial transcriptomics, can resolve cell-type and tissue-region specificity [56,71,72]. Candidate ranking should nevertheless account for sparse single-cell data, biological replication, pseudo-bulk consistency, and known marker genes [73].

### 5.4. Integrated Prioritization for Functional Testing

Candidates should advance to functional testing when several independent lines of evidence are concordant in the relevant tissue or developmental stage. Priority is increased by a credible target gene, allele-specific effects, and agreement among genetic, chromatin, and expression data. By contrast, a candidate supported by only one epigenetic feature, an uncertain target gene, or weak tissue specificity should remain at the annotation stage [27,54,55,56].

## 6. Causal Functional Validation of Negative Regulatory Features

**Figure 2:**
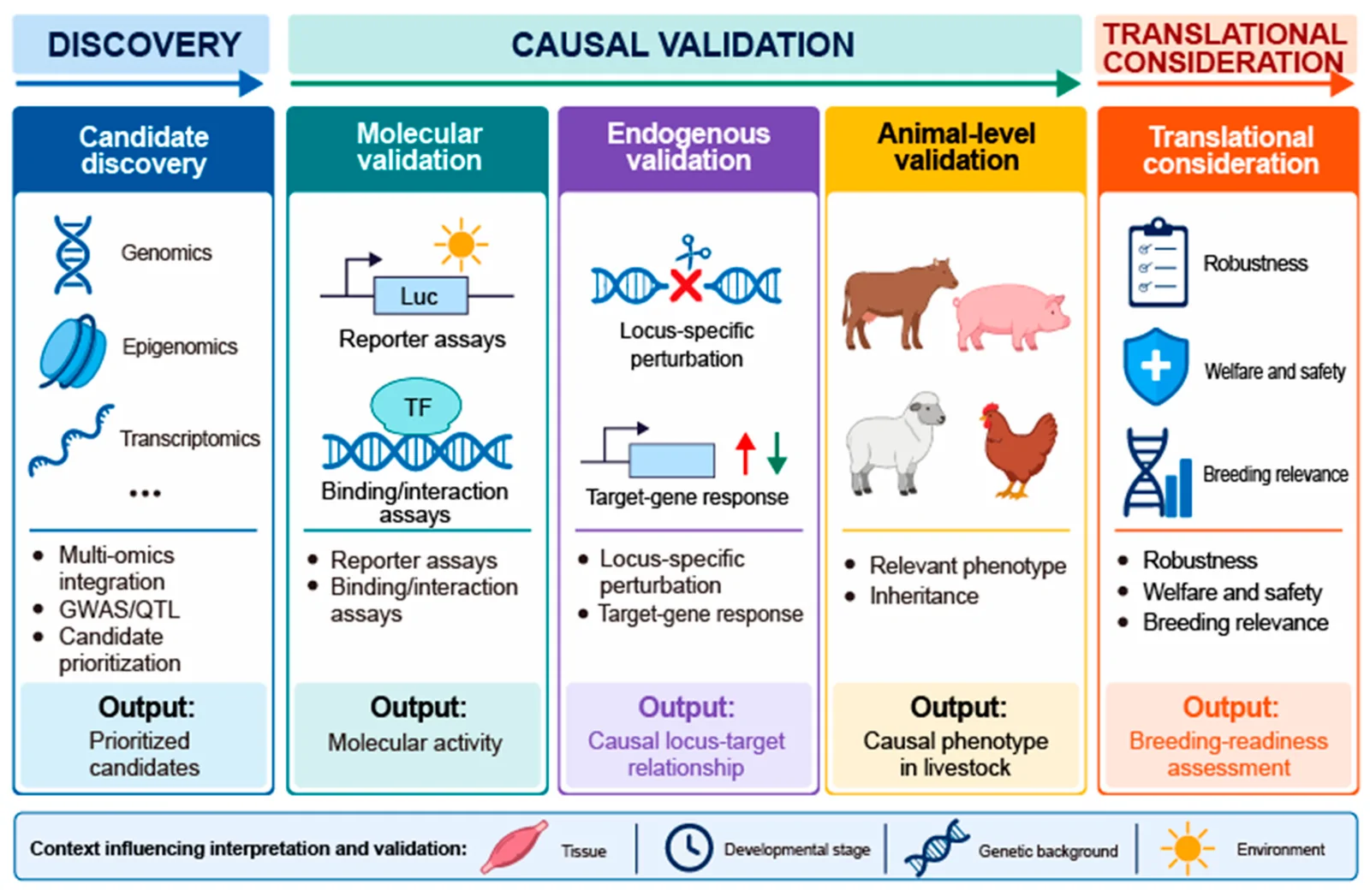
Workflow for discovery, causal validation, and translational consideration of negative regulatory features in livestock. The red upward and green downward arrows indicate increased and decreased target-gene expression, respectively. Abbreviations: GWAS, genome-wide association study; QTL, quantitative trait locus; TF, transcription factor; Luc, luciferase.

Causal validation is the central step that distinguishes a negative regulatory feature from a correlated genomic signal. Experiments should be tailored to the molecular class of the candidate and should connect regulatory activity, the endogenous target-gene response, and a biologically relevant phenotype. Figure 2 summarizes the progression from candidate discovery to molecular testing, endogenous causality, and animal-level validation; downstream breeding relevance should be considered only after this evidentiary chain is established.

### 6.1. Reporter Assays and Allele-Specific Effect Testing

Dual-luciferase reporter assays can compare the activity of a candidate fragment with that of a control construct and can test allele-specific effects by placing alternative alleles in otherwise identical vectors. Massively parallel reporter assays (MPRAs) use sequence barcodes to assay large numbers of candidate fragments and alleles in parallel and are therefore suitable for screening candidates derived from GWAS, QTL, and multi-omics analyses [74]. Self-transcribing active regulatory region sequencing (STARR-seq) enables large-scale activity profiling, although the classical assay was developed mainly for enhancer discovery [75]. When adapted to sequence-defined negative regulatory elements, the reporter system should have stable basal activity and include appropriate positive and negative controls.

Reporter assays provide high throughput but generally lack the endogenous chromatin modifications, genomic position, and three-dimensional contacts of the native locus. The same sequence can display different activity when assayed episomally and after chromosomal integration [76]. Reporter assays should therefore be interpreted as evidence of repressive potential and followed by endogenous testing of the candidate locus and its predicted target gene.

### 6.2. Transcription Factor Binding and Chromatin Interactions

Electrophoretic mobility shift assay (EMSA) can assess protein-DNA binding in vitro, and specificity can be tested using competitor probes, motif-mutant probes, and antibody supershift assays [77]. Chromatin immunoprecipitation followed by quantitative PCR (ChIP-qPCR) provides targeted validation of factor or histone-mark enrichment at a candidate locus. Cleavage under targets and tagmentation (CUT&Tag) can profile transcription factors, co-repressors, and histone modifications from small samples with relatively low background [78].

For distal candidates, chromosome conformation capture (3C) measures contact frequency between two selected genomic regions, whereas Capture-C enables high-resolution analysis of interactions between candidate elements and one or more promoters [79,80]. These approaches can support a proposed transcription-factor mechanism or element-target connection, but they should be interpreted together with target-gene responses after endogenous perturbation.

### 6.3. Endogenous Locus Perturbation

Endogenous perturbation is essential for testing candidate function in its native chromatin context. Regions with relatively well-defined boundaries can be assessed by dual-single-guide RNA (sgRNA) deletion, whereas core-motif mutation or allele replacement is preferable when activity is concentrated within a small number of nucleotides. In situ saturation mutagenesis can identify critical functional bases within a candidate region [81]. Once a functional single-nucleotide or short-sequence variant has been defined, base editing or prime editing can reconstruct the corresponding regulatory allele while minimizing changes to the surrounding sequence.

Large deletions may disrupt neighboring elements, alter regulatory distance, or reorganize local chromatin. Functional conclusions should therefore be reproduced with independent sgRNA pairs or orthogonal editing strategies, and edited loci should be examined for unintended large deletions, inversions, and rearrangements. CRISPR interference (CRISPRi) and other CRISPR-based epigenome-editing approaches can modify chromatin and transcriptional states without permanently changing the DNA sequence [82,83]. For a candidate silencer, however, additional recruitment of Krüppel-associated box (KRAB) increases repression rather than removing the native function and therefore does not constitute a rigorous loss-of-function test. In situ deletion, mutation of a core repressive motif, or allele replacement provides more direct evidence of sequence dependence.

### 6.4. Rescue Experiments and Directional Consistency

A change in target-gene expression or cellular phenotype after candidate perturbation does not completely exclude off-target effects, damage to adjacent sequences, or secondary pathway responses. Rescue experiments can test pathway specificity by restoring the wild-type sequence, re-establishing the original regulatory state, or counter-regulating the target gene.

For example, if deletion of a candidate silencer increases target-gene expression, subsequent reduction in that target gene can determine whether the cellular or animal phenotype is rescued. Allelic studies can also use reciprocal replacement to test whether alternative alleles produce opposite molecular effects. In a mouse model, CRISPR-mediated activation of a promoter or enhancer restored gene expression and associated phenotypes in vivo [84], providing a methodological precedent for rescue experiments involving cis-regulatory regions rather than direct livestock evidence. A strong causal chain should show directional consistency among the regulatory sequence, factor binding or chromatin state, target-gene expression, and biological phenotype.

### 6.5. Animal-Level and Germline Transmission Validation

Cellular and embryonic experiments can demonstrate effects on endogenous expression but cannot substitute for validation in animals. Founder-generation (F0) animals produced by zygote editing may be mosaic and may carry multiple edited alleles, with edit composition varying among tissues [85,86,87]. Genotyping should therefore include multiple tissues and should specifically quantify allele identity, edit proportion, and target-gene expression in the biologically relevant tissue, rather than relying solely on blood, ear, or skin samples.

Animal-level validation should also establish directional consistency between molecular and phenotypic effects. Disruption of a candidate silencer or repressive motif should derepress the predicted target gene in the relevant tissue and should be followed by measurement of biologically related growth, developmental, metabolic, or reproductive phenotypes. To reduce ambiguity caused by F0 mosaicism and heterogeneous edited alleles, analyses should use animals with well-defined genotypes, appropriate controls, adequate sample sizes where feasible, and independent editing cohorts.

Once the expected molecular and phenotypic effects have been observed in F0 animals, breeding is required to confirm germline transmission and stable inheritance. Offspring analysis can separate distinct F0 alleles and enable molecular and phenotypic validation in a more clearly defined genotypic background [88]. Tier 4 assignment requires a defined edited or naturally segregating allele, a molecular response in the relevant target tissue, and a reproducible animal phenotype. Germline transmission or inherited segregation provides stronger support where applicable.

## 7. Considerations for Translation After Causal Validation

Functional annotation, causal validation, and breeding suitability represent distinct levels of evidence and should not be treated as interchangeable. Identification of a regulatory candidate should not be interpreted as evidence that it is suitable for genomic selection or genome editing. Candidate discovery should first be followed by molecular functional testing, endogenous causal validation, and, where relevant, animal-level validation before breeding application is considered.

For a causally supported regulatory allele, translational evaluation should consider effect size, allele frequency in relevant populations, tissue and developmental specificity, genetic-background dependence, pleiotropic effects, inheritance, production relevance, and stability across environments. Natural alleles already segregating in livestock populations may be prioritized for selection only when their effects are reproducible and relevant to the breeding objective, whereas functional annotations can support candidate prioritization and genomic-prediction models rather than serve as direct evidence of breeding value [89,90,91,92,93].

Genome editing should therefore be viewed primarily as a tool for locus-specific causal validation and, only after sufficient evidence has accumulated, as a potential route for reconstructing a validated regulatory allele. The porcine ZBED6–IGF2 locus illustrates how precise editing can connect a defined non-coding sequence with target-gene expression, animal phenotype, and inheritance [29,30]. Technical editing feasibility alone does not establish breeding suitability. Any proposed application would additionally require evaluation of genomic integrity, unintended or off-target consequences, phenotypic robustness, stable inheritance, animal health and welfare, product safety, and relevant regulatory considerations [94,95,96,97,98].

## 8. Limitations

Several limitations should be considered when interpreting the evidence summarized in this review. First, this is a narrative rather than a systematic review or meta-analysis, and the livestock examples were selected to illustrate major regulatory classes and different levels of functional evidence rather than to provide a quantitative representation of the entire literature. The available evidence is also uneven across species, tissues, traits, and regulatory mechanisms. In particular, many livestock studies remain at the levels of candidate annotation or molecular functional evidence, whereas relatively few loci have been validated through endogenous perturbation, animal phenotyping, and inheritance testing. In addition, evidence from human or model-organism systems was used to inform general mechanistic or methodological principles but was not considered equivalent to direct livestock evidence when evaluating evidence strength.

A further limitation is the insufficient characterization of environmental and genotype-by-environment (G × E) effects on regulatory variation in livestock. G × E is well documented for economically important traits in cattle, including growth, reproduction, milk production, heat tolerance, disease resistance, and feed efficiency, indicating that genetic effects may differ across production environments [99]. Environmental stress can also influence gene expression and epigenetic regulation; for example, heat exposure has been associated with persistent phenotypic and epigenetic effects in several livestock species [100]. These observations raise the possibility that the magnitude or direction of a negative regulatory effect may depend on thermal, nutritional, physiological, or management conditions. Consequently, a regulatory locus validated under one experimental or production environment should not automatically be assumed to exert the same molecular or phenotypic effect across breeds or environments. Future studies should therefore incorporate multi-environment validation and, where feasible, longitudinal designs to assess the robustness of regulatory effects before breeding application is considered.

## 9. Conclusions and Future Directions

Negative regulatory features constitute an important but still incompletely validated component of livestock functional genomics. Although functional annotation, multi-omics integration, and high-resolution regulatory mapping are rapidly expanding the number of candidate regions [25,26,27,55,66,101,102,103], among the livestock studies evaluated here, evidence is concentrated mainly at the levels of candidate annotation or molecular functional testing, with comparatively fewer loci supported by endogenous and animal-level causal validation. The central challenge is therefore to convert prioritized candidates into experimentally supported regulatory relationships. Future studies should emphasize locus-specific perturbation, direct target-gene validation, relevant animal phenotyping, and inheritance testing [81,84,88,104], while accounting for tissue, developmental, genetic-background, and environmental context.

Candidate regulatory annotation should be regarded as a starting point rather than an endpoint. Endogenous causal validation is required before negative regulatory variants can be credibly considered for livestock breeding applications.

## Figures and Tables

**Table 1:** Database-specific search themes and numbers of records retrieved.

Search Theme	PubMed	Web of Science	Scopus	Total Records
1. Negative regulatory elements/silencers + livestock	172	232	267	671
2. miRNA-binding sites + livestock	93	109	119	321
3. lncRNA-associated negative regulation + livestock	88	90	214	392
4. Repressive chromatin/epigenetic repression + livestock	192	334	453	979
Total records retrieved	545	765	1053	2363

**Table 2:** Study-selection stages and numbers of records.

Study-Selection Stage	Number of Records
Records identified from PubMed	545
Records identified from Web of Science	765
Records identified from Scopus	1053
Total records identified	2363
Duplicate records removed	1169
Unique records after deduplication	1194
Records screened by title and abstract	1194
Records excluded after title/abstract screening	556
Articles assessed for eligibility	638
Articles excluded during eligibility assessment	278
Studies retained for the final narrative synthesis	360

**Table 3:** Classification and minimum evidence requirements for negative regulatory features in livestock genomes.

Type	Defined DNA Sequence	Primary Regulatory Level	Evidence Requirements	Refs
Silencer	Yes	Transcriptional	Candidate: sequence-bounded region with reproducible repressive activity or convergent candidate-level evidence. Endogenous causality: native-locus sequence perturbation changes the predicted target-gene output.	[9,10]
Context-dependent enhancer-silencer element	Yes	Transcriptional	Candidate: the same defined sequence shows opposite regulatory directions across relevant contexts. Endogenous causality: context-matched native-locus perturbation confirms the predicted regulatory direction.	[9,11]
miRNA-binding site	Yes	Post-transcriptional	Candidate: a defined miRNA-binding site is supported by interaction and/or expression evidence. Endogenous causality: site- or allele-specific perturbation with miRNA manipulation changes endogenous target output in the predicted direction.	[12,13]
lncRNA-associated regulation	Context-dependent	Transcriptional or epigenetic	Candidate: negative regulation is associated with an lncRNA locus, transcriptional process, or mature RNA. Endogenous causality: distinct perturbations identify which molecular layer drives the endogenous effect.	[14,15]
Candidate repressive chromatin region	Not necessarily	Epigenetic	Candidate: a region shows reproducible repressive chromatin enrichment and/or reduced transcription. Endogenous causality: targeted manipulation of the relevant epigenetic state or critical sequence changes endogenous target-gene expression.	[16,17]

Note: References in this table include both livestock studies and evidence from human or model-organism systems used to define general regulatory concepts and methodological principles. Model-organism evidence was used for conceptual or mechanistic support only and was not considered equivalent to direct livestock evidence when assigning evidence tiers.

**Table 4:** Evidence tiers for functional evaluation of negative regulatory features in livestock.

Evidence Tier	Operational Definition	Minimum Criteria for Assignment	Representative Approaches
Tier 1: Candidate annotation	A genomic region, sequence, RNA-associated feature, or chromatin state prioritized as a potential negative regulatory feature.	At least one reproducible candidate-level association or annotation links the feature to reduced expression, repressive chromatin, regulatory sequence characteristics, QTL/GWAS signals, or a predicted target. Direct functional perturbation is not required.	ATAC-seq, ChIP-seq/CUT&Tag, WGBS, RNA-seq, motif prediction, GWAS/QTL mapping, multi-omics integration
Tier 2: Molecular functional evidence	Direct evidence that the candidate can exert or participate in a negative regulatory effect at the molecular level.	Candidate-specific molecular activity must be demonstrated by at least one direct functional or interaction assay with appropriate controls, and the assayed sequence, RNA interaction, protein binding, or chromatin effect must be linked to the proposed regulatory mechanism.	Reporter assays, EMSA, ChIP-qPCR, RNA pull-down, miRNA gain- and loss-of-function assays, RNA interference, chromatin-interaction assays
Tier 3: Endogenous causal evidence	Causal negative regulatory function demonstrated in the endogenous cellular or genomic context.	A locus-, allele-, sequence-, RNA-, or epigenetic-state-specific perturbation must produce the predicted change in endogenous target-gene expression or regulatory state, with direct support for target assignment. Rescue or an orthogonal perturbation strengthens, but is not mandatory for, classification.	CRISPR deletion, motif mutation, allele replacement, CRISPRi/a, targeted methylation/demethylation, epigenome editing, selective RNA or transcriptional perturbation
Tier 4: Animal-level causal evidence	A causal regulatory effect linked to a biologically relevant phenotype in livestock.	The regulatory allele or perturbation must be validated in animals, with evidence connecting genotype or perturbation to the molecular regulatory consequence and a relevant phenotype. Independent replication, germline transmission, or offspring validation provides stronger support where applicable.	Edited animals, naturally segregating causal alleles, animal-level genotype–phenotype analysis, offspring or inheritance validation

Note: Tier assignment reflects the highest level of evidence directly demonstrated in each study. The tiers do not represent an obligatory linear progression, and evidence from multiple lower-tier observations does not, by itself, satisfy the defining criterion of a higher tier. Experimental requirements are mechanism-dependent: sequence-defined silencers require sequence-specific functional testing; miRNA-binding sites require interrogation of the binding site and/or miRNA activity; lncRNA-associated regulation may require separation of DNA-locus, transcriptional, and mature-RNA effects; and candidate repressive chromatin regions require targeted manipulation of the relevant epigenetic state. Tier 4 represents animal-level causal evidence rather than evidence of breeding readiness.

**Table 5:** Illustrative livestock negative regulatory features and candidate regions evaluated using the four-tier evidence framework.

Species/Trait	Feature Class	Regulatory Feature/Locus	Tissue/Cell Type and Context	Methods and Main Evidence	Evidence Tier	Ref.
Dairy cattle/milk fatty-acid composition	miRNA-binding site	FADS2 c.1571G>A/miR-744	Mammary epithelial cells; lactation	Association + reporter + miR-744 gain/loss + mRNA/protein assays; A allele weakens miR-744 binding.	Tier 2	[45]
Dairy cattle/mastitis	miRNA-binding site	NCF4 g.18475A>G/bta-miR-2426	Mammary tissue; mastitis/SCS context	Association + reporter + RT-qPCR; allele alters miRNA binding and NCF4 expression.	Tier 2	[46]
Dairy cattle/milk and carcass traits	miRNA-binding site	OLR1 3′ UTR variant/miR-370	Dairy and beef cattle trait context	Reporter and expression analyses; variant diminishes miR-370 binding and alters OLR1 expression.	Tier 2	[47]
Sheep/wool crimp	miRNA-binding site	DLX3 3′ UTR indel/miR-188	Sheep fetal fibroblasts; wool phenotype	Reporter + miR-188 mimic/inhibitor + endogenous expression; allele-dependent repression.	Tier 2	[48]
Sheep/meat production	miRNA-binding site	MAFA g.1618G>A/miR-3678-3p	Longissimus dorsi; lambs and ovine myoblasts	Genotype–phenotype association + reporter + protein + myoblast/ChIP assays; associated with longissimus muscle proportion and meat production.	Tier 4	[49]
Cattle/adipogenesis	lncRNA-associated negative regulation	BADLNCR1–GLRX5	Bovine adipocytes	Knockdown + reporter + ChIP/CHIRP + GLRX5 rescue; BADLNCR1 suppresses adipogenesis.	Tier 2	[50]
Cattle/adipogenesis	lncRNA-associated negative regulation	MIR221HG	Bovine adipose-derived stem cells	siRNA + RT-qPCR/protein + Oil Red O; MIR221HG knockdown promotes adipogenesis.	Tier 2	[51]
Cashmere goat /hair-follicle cycle	Candidate repressive chromatin region	H19 and HOTAIR promoter methylation	Secondary hair follicles; anagen/catagen/telogen	BSP + expression analyses; promoter methylation is associated with lower lncRNA expression; no targeted perturbation.	Tier 1	[52,53]
Texel sheep/muscularity	miRNA-binding site	MSTN 3′ UTR G>A/miR-1/miR-206	Skeletal muscle; inherited muscularity	Natural causal allele + allele-dependent miRNA assays; inherited muscular hypertrophy in Texel sheep.	Tier 4	[13]
Pig/muscle and meat quality	Sequence-defined cis-regulatory motif	ZBED6 motif in IGF2 intron 3	Skeletal muscle; edited pigs and offspring	CRISPR/Cas9 + base editing + expression + animal/offspring phenotyping; increased muscle growth and altered carcass/meat-quality traits.	Tier 4	[29,30]

Note: The cases shown are illustrative rather than comprehensive. Additional examples were included to broaden species, trait contexts, and regulatory mechanisms. Tier assignment reflects the highest level of evidence directly demonstrated in each study and should not be interpreted as a quantitative estimate of the entire livestock literature.

## Data Availability

No new data were created or analyzed in this study. Data sharing is not applicable to this article.

## Supplementary Materials

The following supporting information can be downloaded at: https://www.mdpi.com/article/10.3390/ani16182956/s1, Table S1: Complete database-specific search strategies used in PubMed, Web of Science Core Collection, and Scopus; Table S2: Study-selection process and numbers of records; Table S3: Eligibility and representative-study selection criteria.
