# Supplementary material for: Non-Coding Negative Regulatory Features in Livestock Genomes: Functional Annotation and Causal Validation
Source: Animals (Basel). 2026 Sep 20;16(18):2956. doi: 10.3390/ani16182956 (PMC13603999; doi:10.3390/ani16182956)
Supplement: Supplementary file 1 [file animals-16-02956-s001.zip › animals-4553483-supplementary.pdf]

Supplementary Materials

Non-coding negative regulatory features in livestock genomes: from functional annotation and causal validation to regulome-informed breeding

Review design and search period. This study was conducted as a structured narrative review. Searches were performed in PubMed, Web of Science Core Collection, and Scopus. No lower publication-year limit was imposed, and the final searches were completed on 4 September 2026. The same four conceptual search themes were used across databases, with syntax adapted to the requirements of each platform. No separate MeSH restriction was applied in PubMed; the PubMed searches were based on Title/Abstract terms.

Supplementary Table S1. Complete database-specific search strategies used in PubMed, Web of Science Core Collection, and Scopus

Search terms were grouped into four themes. Species terms were restricted to major livestock and poultry species relevant to the review. Counts are the records retrieved at the final search on 4 September 2026, before cross-database deduplication.

Database	Search theme	Search string	Records retrieved
PubMed	1. Negative regulatory elements / silencers	("negative regulatory element"[Title/Abstract] OR "negative regulatory elements"[Title/Abstract] OR silencer[Title/Abstract] OR silencers[Title/Abstract] OR "transcriptional silencer"[Title/Abstract] OR "transcriptional silencers"[Title/Abstract] OR "repressor element"[Title/Abstract] OR "repressor elements"[Title/Abstract] OR "repressive regulatory element"[Title/Abstract] OR "repressive regulatory elements"[Title/Abstract]) AND (livestock[Title/Abstract] OR cattle[Title/Abstract] OR bovine[Title/Abstract] OR sheep[Title/Abstract] OR ovine[Title/Abstract] OR goat[Title/Abstract] OR caprine[Title/Abstract] OR pig[Title/Abstract] OR porcine[Title/Abstract] OR swine[Title/Abstract] OR chicken[Title/Abstract] OR poultry[Title/Abstract])	172
PubMed	2. miRNA-binding sites	("miRNA-binding site"[Title/Abstract] OR "miRNA-binding sites"[Title/Abstract] OR "microRNA-binding site"[Title/Abstract] OR "microRNA-binding sites"[Title/Abstract] OR "miRNA target site"[Title/Abstract] OR "miRNA target sites"[Title/Abstract] OR "microRNA target site"[Title/Abstract] OR "microRNA target sites"[Title/Abstract]) AND (livestock[Title/Abstract] OR cattle[Title/Abstract] OR bovine[Title/Abstract] OR sheep[Title/Abstract] OR ovine[Title/Abstract] OR goat[Title/Abstract] OR caprine[Title/Abstract] OR pig[Title/Abstract] OR porcine[Title/Abstract] OR swine[Title/Abstract] OR chicken[Title/Abstract] OR poultry[Title/Abstract])	93
PubMed	3. lncRNA-associated negative regulation	(lncRNA[Title/Abstract] OR "long non-coding RNA"[Title/Abstract] OR "long noncoding RNA"[Title/Abstract]) AND (repress*[Title/Abstract] OR silencing[Title/Abstract] OR "negative regulation"[Title/Abstract]) AND (livestock[Title/Abstract] OR cattle[Title/Abstract] OR bovine[Title/Abstract] OR sheep[Title/Abstract] OR ovine[Title/Abstract] OR goat[Title/Abstract] OR caprine[Title/Abstract] OR pig[Title/Abstract] OR porcine[Title/Abstract] OR swine[Title/Abstract] OR chicken[Title/Abstract] OR poultry[Title/Abstract])	88
PubMed	4. Repressive chromatin / epigenetic repression	("DNA methylation"[Title/Abstract] OR "repressive chromatin"[Title/Abstract] OR H3K27me3[Title/Abstract] OR H3K9me3[Title/Abstract] OR H4K20me3[Title/Abstract]) AND (repress*[Title/Abstract] OR silencing[Title/Abstract] OR "negative regulation"[Title/Abstract]) AND (livestock[Title/Abstract] OR cattle[Title/Abstract] OR bovine[Title/Abstract] OR sheep[Title/Abstract] OR ovine[Title/Abstract] OR goat[Title/Abstract] OR caprine[Title/Abstract] OR pig[Title/Abstract] OR porcine[Title/Abstract] OR swine[Title/Abstract] OR chicken[Title/Abstract] OR poultry[Title/Abstract])	192
Web of Science Core Collection	1. Negative regulatory elements / silencers	TS=(("negative regulatory element*" OR silencer* OR "transcriptional silencer*" OR "repressor element*" OR "repressive regulatory element*") AND (livestock OR cattle OR bovine OR sheep OR ovine OR goat OR caprine OR pig OR porcine OR swine OR chicken OR poultry))	232
Web of Science Core Collection	2. miRNA-binding sites	TS=(("miRNA-binding site*" OR "microRNA-binding site*" OR "miRNA target site*" OR "microRNA target site*") AND (livestock OR cattle OR bovine OR sheep OR ovine OR goat OR caprine OR pig OR porcine OR swine OR chicken OR poultry))	109
Web of Science Core Collection	3. lncRNA-associated negative regulation	TS=((lncRNA OR "long non-coding RNA" OR "long noncoding RNA") AND (repress* OR silencing OR "negative regulation") AND (livestock OR cattle OR bovine OR sheep OR ovine OR goat OR caprine OR pig OR porcine OR swine OR chicken OR poultry))	90
Web of Science Core Collection	4. Repressive chromatin / epigenetic repression	TS=(("DNA methylation" OR "repressive chromatin" OR H3K27me3 OR H3K9me3 OR H4K20me3) AND (repress* OR silencing OR "negative regulation") AND (livestock OR cattle OR bovine OR sheep OR ovine OR goat OR caprine OR pig OR porcine OR swine OR chicken OR poultry))	334
Scopus	1. Negative regulatory elements / silencers	TITLE-ABS-KEY("negative regulatory element*" OR silencer* OR "transcriptional silencer*" OR "repressor element*" OR "repressive regulatory element*") AND TITLE-ABS-KEY(livestock OR cattle OR bovine OR sheep OR ovine OR goat OR caprine OR pig OR porcine OR swine OR chicken OR poultry)	267
Scopus	2. miRNA-binding sites	TITLE-ABS-KEY("miRNA-binding site*" OR "microRNA-binding site*" OR "miRNA target site*" OR "microRNA target site*") AND TITLE-ABS-KEY(livestock OR cattle OR bovine OR sheep OR ovine OR goat OR caprine OR pig OR porcine OR swine OR chicken OR poultry)	119

Scopus	3. lncRNA-associated negative regulation	TITLE-ABS-KEY(lncRNA OR "long non-coding RNA" OR "long noncoding RNA") AND TITLE-ABS-KEY(repress* OR silencing OR "negative regulation") AND TITLE-ABS-KEY(livestock OR cattle OR bovine OR sheep OR ovine OR goat OR caprine OR pig OR porcine OR swine OR chicken OR poultry)	214
Scopus	4. Repressive chromatin / epigenetic repression	TITLE-ABS-KEY("DNA methylation" OR "repressive chromatin" OR H3K27me3 OR H3K9me3 OR H4K20me3) AND TITLE-ABS-KEY(repress* OR silencing OR "negative regulation") AND TITLE-ABS-KEY(livestock OR cattle OR bovine OR sheep OR ovine OR goat OR caprine OR pig OR porcine OR swine OR chicken OR poultry)	453

Database totals: PubMed, 545; Web of Science, 765; Scopus, 1053; total records retrieved before deduplication, 2363.

Supplementary Table S2. Study-selection process and numbers of records

Records from all databases and search themes were merged before deduplication. Duplicate records were identified using DOI, PMID, and normalized title matching, with manual verification of near-identical title variants. Because this was a structured narrative review rather than a systematic review, the post-screening stage was recorded as detailed eligibility screening rather than formal PRISMA-style full-text screening.

Study-selection stage	Number of records
Records identified from PubMed	545
Records identified from Web of Science	765
Records identified from Scopus	1053
Total records identified	2363
Duplicate records removed	1169
Unique records after deduplication	1194
Records screened by title and abstract	1194
Records excluded after title/abstract screening	556
Records retained for detailed eligibility screening	638
Records excluded after detailed eligibility screening	278
Records retained after detailed eligibility screening	360

Supplementary Table S3. Eligibility and representative-study selection criteria

Criterion	Operational definition
Population / species	Primary emphasis on livestock and poultry species, including pigs, cattle, sheep, goats, and chickens/poultry. Model-organism studies were retained only when they provided directly relevant mechanistic or methodological evidence and were clearly distinguished from livestock evidence.
Regulatory scope	Studies addressing sequence-defined silencers or repressor elements, context-dependent negative regulatory elements, functional miRNA-binding sites, lncRNA-associated negative regulation, or candidate repressive chromatin linked to transcriptional repression.
Eligible evidence	Candidate annotation, multi-omics association, reporter assays, binding/occupancy evidence, endogenous perturbation, genome/epigenome editing, target-gene validation, animal phenotyping, or inheritance evidence relevant to negative regulation.
Exclusion criteria	Studies were excluded when they were unrelated to negative regulation; concerned non-livestock species without directly relevant mechanistic value; used “silencing” only to denote generic siRNA/shRNA knockdown; were unrelated clinical/pharmacological studies; or lacked sufficient relevance to the defined regulatory categories.
Representative-study selection	Representative studies were prioritized when they (i) directly addressed a predefined negative-regulatory category, (ii) involved livestock or provided uniquely informative mechanistic evidence, (iii) contained clear experimental or multi-omics support, (iv) contributed non-redundant information across species, tissues, developmental stages, or evidence tiers, and (v) allowed assignment within the four-tier evidence framework.
Evidence-tier use	Tier 1: candidate annotation; Tier 2: molecular functional evidence; Tier 3: endogenous causal evidence; Tier 4: animal-level causal evidence. Tier assignment was based on the strongest evidence reported for the specific regulatory feature rather than the overall technical complexity of the study.
